# Optical coherence tomography angiography microvascular findings in macular edema due to central and branch retinal vein occlusions

**DOI:** 10.1038/srep40763

**Published:** 2017-01-18

**Authors:** Mastropasqua Rodolfo, Toto Lisa, Di Antonio Luca, Borrelli Enrico, Senatore Alfonso, Di Nicola Marta, Di Martino Giuseppe, Ciancaglini Marco, Paolo Carpineto

**Affiliations:** 1 Ophthalmology Clinic, Moorfields Eye Hospital, London, EC1V 2PD, UK; 2 Ophthalmology Clinic, Department of Medicine and Science of Ageing, University G. D’Annunzio Chieti-Pescara, Chieti, 66100, Italy; 3 Department of Experimental and Clinical Sciences, Laboratory of Biostatistics, University “G. d’Annunzio” Chieti-Pescara, Chieti, 66100, Italy; 4 Ophthalmology Clinic, University of l’Aquila, Aquila, 67100, Italy

## Abstract

The aim of this study was to evaluate retinal and choriocapillaris vessel density using optical coherence tomography angiography (OCTA) in eyes with central retinal vein occlusion (CRVO) and branch retinal vein occlusion (BRVO) complicated by macular edema (ME). Sixty eyes of 60 patients with CRVO or BRVO and ME and 40 healthy subjects underwent measurements of superficial and deep foveal and parafoveal vessel density (FVD, PFVD) and choricapillary density using OCTA at baseline and 60 days after intravitreal dexamethasone implant (IVDEX). FVD and PFVD of the superficial plexus were not significantly lower in CRVO group compared to the controls while in the BRVO group overall PFVD were significantly lower compared to control group (p < 0.001). Overall PFVD of the deep plexus was significantly lower in CRVO and BRVO groups compared to the control group (p < 0.001). FVD and overall PFVD of choriocapillaris were significantly reduced compared to controls in CRVO group (p < 0.001) and PFVD of choriocapillaris was significantly reduced compared to controls in the affected hemi fields in BRVO groups (p < 0.001). OCTA showed vessel density reduction in BRVO and CRVO with main involvement of the deep retinal plexus compared to the superficial retinal plexus due to ischemia that did not recover after intravitreal dexamethasone implant.

Retinal vein occlusions (RVOs) are the second most common retinal vascular disease after diabetic retinopathy^1^.

Among sight threatening complications of RVOs there are macular edema and retinal non-perfusion. Fluorescein angiography (FA) is useful for identifying retinal ischemia and macular edema characterized by dye leakage in the late phases of FA angiograms^2^.

Enhanced imaging system such as adaptive optics (AO) scanning light ophthalmoscope (SLO) FA have been used to better characterize retinal microvasculature in vascular retinal diseases such as RVO with vascular density evaluation^3^,^4^.

Recently optical coherence tomography angiography (OCTA), a non-invasive imaging technique, has been conceived allowing the study of retinal microvasculature without dye injection^5^,^6^,^7^. The movement of red blood cells (RBC) within the retinal capillaries is used as an intrinsic contrast medium to generate flow imaging. OCTA provides non-invasive imaging of the superficial and deep retinal capillary network with detailed foveal avascular zone (FAZ) visualization and vessel density evaluation useful in different vascular diseases^5^.

Some authors investigated the OCTA features of patients with RVO^8^,^9^,^10^. Coscas *et al*. observed retinal capillary network abnormalities in all patients in both superficial capillary plexus and deep capillary plexus on OCTA with detection of ischemic areas more frequent in the deep capillary plexus^9^.

Samara *et al*. investigated superficial and deep retinal capillary densities in patients affected by branch retinal vein occlusion (BRVO) and reported lower vascular densities both in the superficial and deep plexus with correlation between visual function and FAZ area and vessel density^10^.

In the current study, two aspects not previously reported in the literature were explored in eyes affected by RVOs using an OCTA with a Split-Spectrum Amplitude Decorrelation Algorithm (SSADA) such as modification of vessel density after intravitreal dexamethasone implant (IVDEX) and choriocapillaris density compared to that of eyes of healthy age-matched subjects.

## Results

### Demographic data

Three out of 63 patients were excluded from the study for poor image quality of OCTA images.

The mean age was 58.7 ± 17.6 years for patients with CRVO, 59.3 ± 5.6 years for patients with BRVO supero-temporal (ST), 55.9 ± 6.3 years for patients with BRVO infero-temporal (IT) and 56.1 ± 14.3 for the control group. The difference in age between the four groups was not statistically significant (p = 0.416). In the overall RVOs group of 60 patients 48.3% were females and 51.7% patients were males and in the control group of 40 subjects 52.6% were females and 47.4% were males (p = 0.729). Among RVOs 20 eyes were affected by CRVO and 40 eyes by BRVO (20 superotemporal BRVO, 20 inferotemporal BRVO).

At FA 2 out of 20 eyes with CRVO and 18 out of 40 eyes with BRVO showed macular ischemia (p = 0.015).

Four eyes in the CRVO group and 10 eyes in the BRVO group had received prior treatment anti–vascular endothelial growth factor (VEGF) injections while the remaining patients (46) were treatment naïve (p = 0.804).

### Visual acuity and microperimetry

The mean BCVA and microperimetry (4°, 8°, 20°) values are reported in Table 1. The mean BCVA and microperimetry are significantly lower in the RVOs patients compared to controls (p < 0.001 for BCVA, p < 0.001 for microperimtery at 4° and 8°) with lower values in CRVO compared to BRVO ST. Microperimetry within 4° and 8° is significantly lower in CRVO compared to BRVO (Table 1).

The 8° degree sensitivity and the inferior emifield, were significantly influenced by presence of macular ischemia (p = 0.036 and p = 0.020, respectively) with lower microperimetric values in macular ischemic *vs* non macular ischemic eyes at FA.

### Quantitative vessel density analysis

Whole enface, FVD and PFVD of the superficial, deep plexus and of the choriocapillaris are shown in Table 2. Whole enface, FVD and PFVD of the superficial plexus were not significantly lower in CRVO group compared to the control group while in the BRVO group overall PFVD was significantly lower compared to control group with highest statistical significance in the affected sectors (p < 0.001). Whole enface and overall PFVD of the deep plexus were significantly lower in CRVO and BRVO groups compared to the control group (p < 0.001). In CRVO group deep PFVD in the superior and inferior hemi fields was significantly lower compared to controls (p < 0.001). In BRVO groups deep PFVD in the superior and inferior hemi fields were respectively reduced in supero- and inferotemporal BRVO (p < 0.001). Whole enface FVD and overall PFVD of choriocapillaris was significantly reduced compared to controls in CRVO group (p < 0.001). PFVD of choriocapillaris was significantly reduced compared to controls in the affected hemi fields in BRVO groups (p < 0.001).

FVD of superficial plexus, PFVD of chorocapillaris and para- superior hemi fields and para-inferior hemi fields were significantly different in CRVO and BRVO groups considering the presence of macular ischemia (Table 2).

### Qualitative vessel analysis

Superficial retinal vessels in the macular area were diffusely rarified in CRVO particularly in eyes with macular ischemia at FA and in the affected sector of both types of BRVO. In presence of macular ischemia disruption of perifoveal anastomotic arcade was a frequent finding.

**Figure 1:**
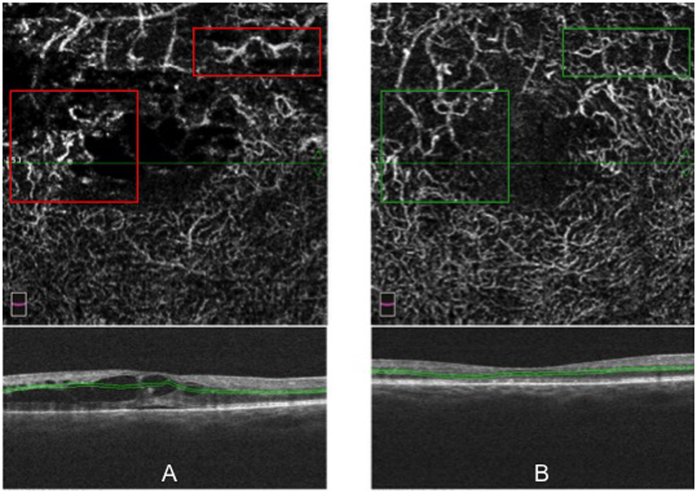
Optical coherence tomography (OCT) angiography images of a patient with supero-temporal branch retinal vein occlusion: deep retinal plexus before dexamethasone implant (panel A) showing areas of telangiectatic vessels (red squares) and after dexamethasone implant (panel B), showing vessel caliber reduction (green squares).

In the deep retinal plexus a rarefaction of vessel density was observed with telengectatic appearance of retinal vessels particularly in the areas of macular edema (Fig. 1). Choriocapillaris showed texture rarefaction in the affected sector of BRVO and in whole macular area in CRVO beneath the retinal edema.

After dexamethasone implant treatment with partial or complete resolution of macular edema deep retinal vessels appeared less telangectatic.

### Retinal macular thickness and subfoveal choroidal thickness analysis

**Figure 2:**
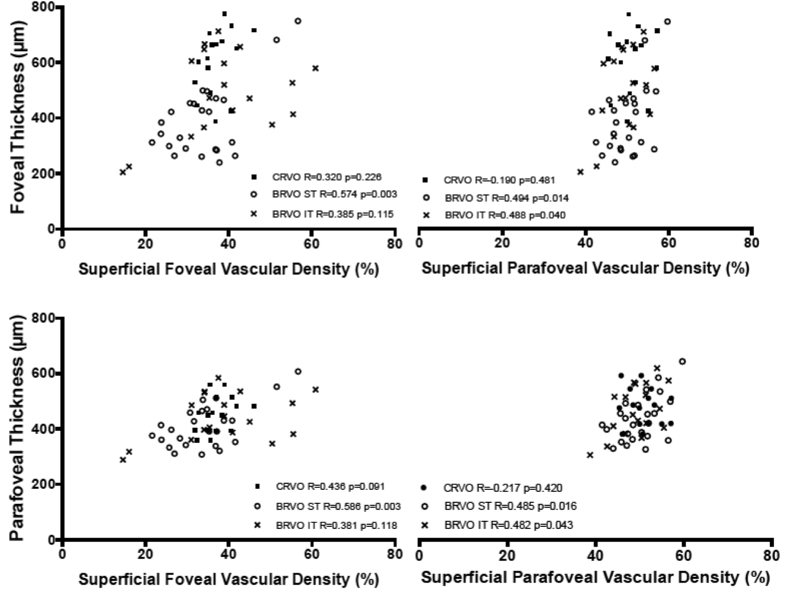
Correlations [Pearson correlation coefficient (R)] between macular thickness (foveal and parafoveal thickness) and superficial vessel density (superficial foveal and parafoveal vascular density) in RVOs patients.

**Figure 3:**
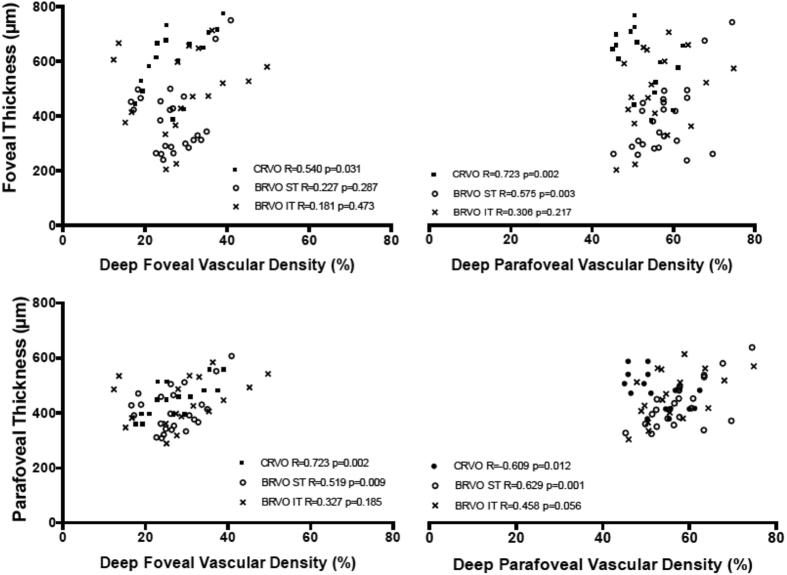
Correlations [Pearson correlation coefficient (R)] between macular thickness (foveal and parafoveal thickness) and deep vessel density (deep foveal and parafoveal vascular density) in RVOs patients.

**Figure 4:**
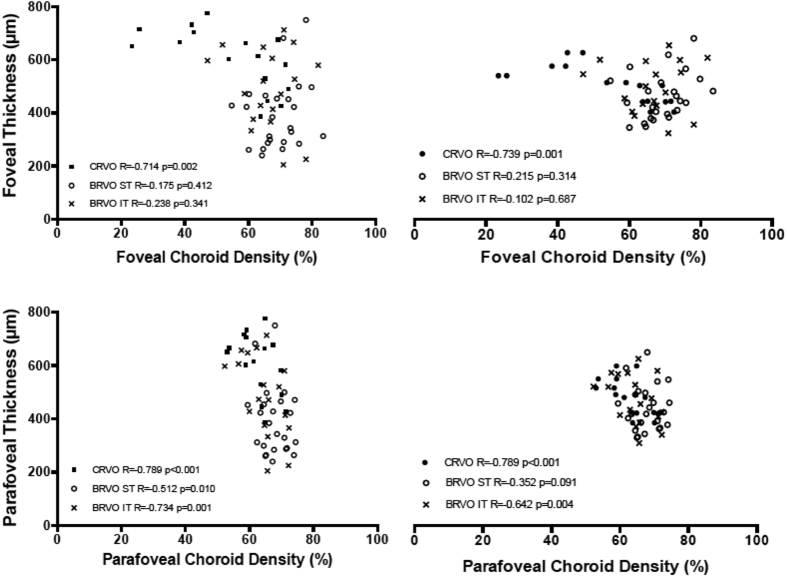
Correlations [Pearson correlation coefficient (R)] between macular thickness (foveal and parafoveal thickness) and choroid vessel density (choroid foveal and parafoveal vascular density) in RVOs patients.

Retinal macular thickness of RVOs patients is significantly increased compared to controls (p < 0.001) (Table S1 in the Supplementary Appendix). Significant correlations were found between macular thickness and visual function (microperimetry and VA) (Figure 1S in the Supplementary Appendix); and between macular thickness and superficial, deep and choriocapillaris vessel density (Figs 2, 3 and 4).

Foveal para-superior and para-inferior macular thickness, were significantly different in CRVO and BRVO groups considering the presence of macular ischemia (p = 0.034, p = 0.001 and p < 0.001, respectively).

Suboveal choroidal thickness was not significantly different between CRVO, BRVO ST and BRVO IT and normal controls (p = 0.672, p = 0.242, p = 0.911) (Table 3) and it was not significantly influenced by presence of macular ischemia at FA.

### Correlation analysis between different parameters

No correlation was found between vessel density and functional parameters. Retinal superficial and deep vessel density (Figs 2 and 3) were positively correlated to foveal and parafoveal retinal thickness on the contrary choriocapillaris density was negatively correlated to foveal and parafoveal retinal thickness (Fig. 4). Functional parameters such as microperimetry and visual acuity (Figure 1S in the Supplementary Appendix) were negatively correlated with foveal and parafoveal thickness.

### Post treatment quantitative analysis

**Figure 5:**
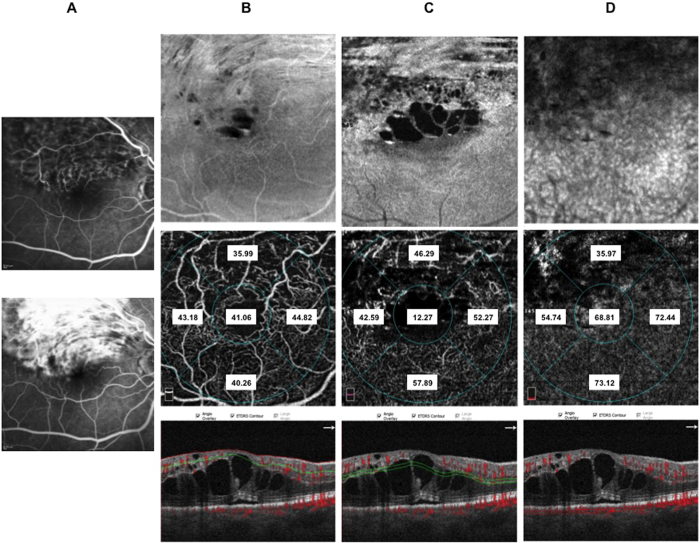
Fluorescein angiography (FA), enface optical coherence tomography (OCT) and OCT angiography (OCTA) images of a patient with ischemic supero-temporal branch retinal vein occlusion. Early (panel A: top) FA and late FA (panel A: bottom) showing tortuous vessels, retinal hemorrhages and diffuse vascular leakage in the late phase. Enface OCT images at the level of the superficial retinal plexus (panel B: top) showing hyporeflectivity in the superior macular area due to retinal edema and hemorrhages and arcuate hypereflectivity due fiber nerve swelling. Corresponding OCTA frame at the level of superficial retinal plexus (panel B: middle) showing rarified vessels in the superior macular area with disruption of perifoveal anastomotic arcade. Enface OCT at the level of the deep retinal plexus (panel C: top) showing numerous hyporeflective central spaces corresponding to retinal cysts separated by Mullers cells pillars, hyporeflective areas due to retinal hemorrhages and arcuate hypereflectivity due fiber nerve swelling. Corresponding OCTA frame at the level of the deep plexus (panel C: middle) showing telangiectatic and rarified vessels at OCTA. Enface OCT at the level of the choriocapillary (panel D: top) showing hyporeflectivity due to overlying retinal edema and hemorrhages. Corresponding OCTA frame at the level of choriocapillaris (panel D: middle) showing apparent reduction of choriocapillaris texture in the area underlying the affected retina. OCTA images with vessel densities and relative longitudinal B-Scan images with highlighted layer segmentation (panel B, C, D: bottom left, bottom middle, bottom right).

**Figure 6:**
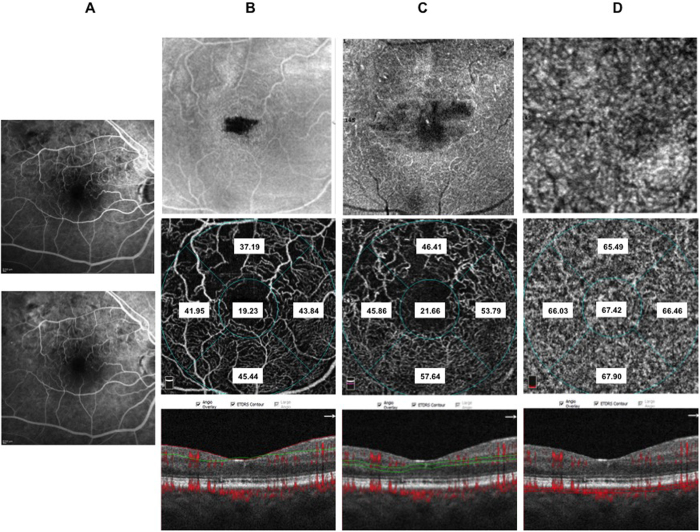
Fluorescein angiography (FA), enface optical coherence tomography (OCT) and OCT angiography (OCTA) images of the same patient after IVDEX implant. Early (panel A: top) FA and late FA (panel A: bottom) reduction of retinal hemorrhages and mild vascular leakage in the late phase. Enface OCT images at the level of the superficial retinal plexus (panel B: top) with almost complete recovery of retinal structure. Corresponding OCTA frame at the level of superficial retinal plexus (panel B: middle) showing rarified vessels in the superior macular area with disruption of perifoveal anastomotic arcade. Enface OCT at the level of the deep retinal plexus (panel C: top) showing partial recovery of retinal structure. Corresponding OCTA frame at the level of the deep plexus (panel C: middle) showing almost normalized vessel caliber that are rarified mainly in the supero-temporal sectors. Enface OCT at the level of the choriocapillary (panel D: top) showing normal texture. Corresponding OCTA frame at the level of choriocapillaris (panel D: middle) showing normal choriocapillaris texture due to resolution of the overlying hemorrhages and intraretinal edema. OCTA images with vessel densities and relative longitudinal B-Scan images with highlighted layer segmentation (panel B, C, D: bottom left, bottom middle, bottom right).

**Figure 7:**
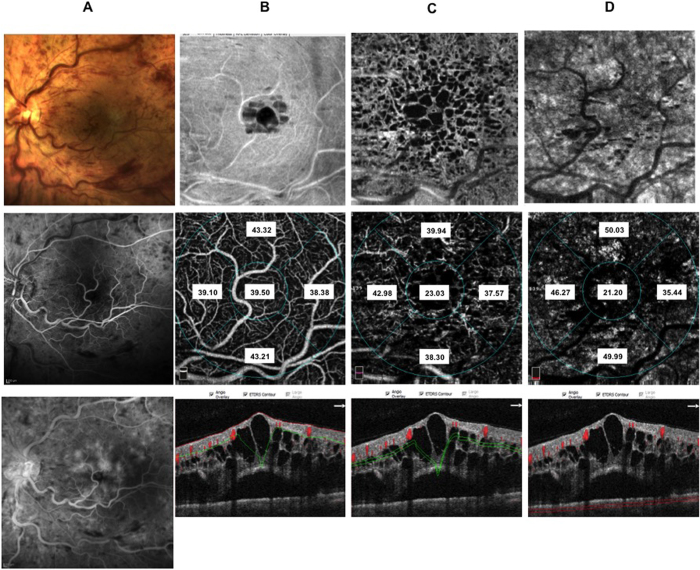
Color fundus, fluorescein angiography (FA), enface optical coherence tomography (OCT) and OCT angiography (OCTA) images of a patient with well perfused central retinal vein occlusion. Color fundus photograph (panel A: top) showing tortuous and congested venous vessels, diffuse retinal hemorrhages and macular edema. Early (panel A: middle) FA and late FA (panel A: bottom) showing tortuous vessels, retinal hemorrhages and vascular leakage with pooling of dye in the late phase. Enface OCT images at the level of the superficial retinal plexus (panel B: middle) showing central hyporeflective spaces due intraretinal cysts. Corresponding OCTA frame at the level of superficial retinal plexus (panel B: top) showing vessels tortuosity. Enface OCT at the level of the deep retinal plexus (panel C: top) showing diffuse hyporeflective spaces corresponding to retinal cysts separated by Mullers cells pillars. Corresponding OCTA frame at the level of the deep plexus (panel C: middle) showing telangiectatic and rarified vessels and flow-void spaces. Enface OCT at the level of the choriocapillary (panel D: top right) showing hyporeflectivity due to overlying retinal edema and hemorrhages. Corresponding OCTA frame at the level of choriocapillaris (panel D: middle right) showing apparent reduction of choriocapillaris texture in the area underlying the affected retina. OCTA images with vessel densities and relative longitudinal B-Scan images with highlighted layer segmentation (panel B, C, D: bottom left, bottom middle, bottom right).

**Figure 8:**
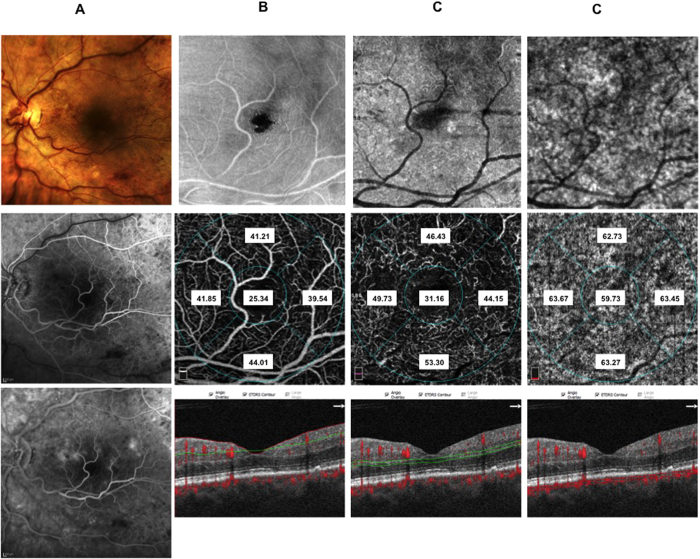
Color fundus, fluorescein angiography (FA), enface optical coherence tomography (OCT) and OCT angiography (OCTA) images of the same patient after IVDEX implant. Color fundus photograph (panel A: top), showing reduction of both hemorrhages and main vessel tortuosity. Early (panel A: middle) FA and late FA (panel A: bottom) reduction of retinal hemorrhages and mild vascular leakage in the late phase. Enface OCT images at the level of the superficial retinal plexus (panel B: top) with complete recovery of retinal structure. Corresponding OCTA frame at the level of superficial retinal plexus (panel B: middle) showing partial reduction of vessel tortuosity. Enface OCT at the level of the deep retinal plexus (panel C: top) showing partial recovery of retinal structure. Corresponding OCTA frame at the level of the deep plexus (panel C: middle) showing almost normalized vessel caliber that appear less dilated. Enface OCT at the level of the choriocapillaris (panel D: top right) showing normal texture. Corresponding OCTA frame at the level of choriocapillaris (panel D: middle right) showing normal choriocapillaris texture due to resolution of the overlying hemorrhages and intraretinal edema. OCTA images with vessel densities and relative longitudinal B-Scan images with highlighted layer segmentation (panel B, C, D: bottom left, bottom middle, bottom right).

Thirty-eight patients (10 CRVO, 12 BRVO ST, 16 BRVO IT) out of 60 patients included in the study underwent IVDEX implant in the vitreous cavity to treat macular edema. In all groups BCVA significantly improved compared to baseline values (Table S2 in the Supplementary Appendix) with significant effect of presence of macular ischemia (p < 0.05) on the treatment. Microperimetry significantly improved in CRVO and BRVO IT and showed a trend toward improvement in BRVO ST (Table S2 in the Supplementary Appendix). Macular thickness of RVOs patients is significantly reduced after treatment (Table S3 in the Supplementary Appendix). Subfoveal choroidal thickness did not change significantly after treatment (Table 4) and it is not significantly influenced by presence of macular ischemia at FA. No significant changes of superficial and deep vessel density was observed after treatment both in CRVO and BRVO on the contrary choriocapillaris density significantly increased in foveal and parafoveal areas in CRVO, in parafoveal area of both BRVO, in foveal area of BRVO ST and in the affected sector of BRVO ST and BRVO IT considering the presence of macular ischemia (Table 5, Figs 5, 6, 7 and 8).

## Discussion

In this study, using OCTA, we investigated retinal superficial and deep vessel densities and choriocapillaris density in patients with both central and branch retinal vein occlusions complicated by macular edema at baseline and after an intravitreal dexamethasone implant. Overall, we found a reduction of foveal and parafoveal retinal superficial and deep vascular density and choriocapillaris density compared to normal controls. Retinal superficial and deep vessel density did not change significantly after intravitreal dexamethasone implant, on the contrary choroid vessel density significantly increased.

FVD and PFVD of the superficial plexus were not significantly lower in CRVO group compared to the control group while in the BRVO group overall PFVD was significantly lower compared to control group with highest statistical significance in the affected sectors (p < 0.001).

Overall PFVD of the deep plexus was significantly lower in CRVO and BRVO groups compared to the control group (p < 0.001). In BRVO groups deep PFVD in the superior and inferior hemi fields were respectively reduced in supero- and inferotemporal BRVO (p < 0.001). FVD and overall PFVD of choriocapillaris was significantly reduced compared to controls in CRVO group (p < 0.001) and PFVD of choriocapillaris was significantly reduced compared to controls in the affected hemi fields in BRVO groups (p < 0.001). The presence of retinal ischemia at FA was related to lower values of superficial and deep vessel density and choriocapillaris density.

The results of our study partially confirm the data from the study of Samara *et al*. that reported lower vascular densities of superficial and deep retinal capillary densities in patients affected by BRVO compared to density values of the fellow eye^10^. In accordance with these results in our study both superficial and deep plexus in BRVO were reduced, nevertheless as already suggested by other authors the deep plexus was more severely affected compared to the superficial plexus. In addition in CRVO the vessel density was reduced only in the deep plexus compared to controls.

The more severe alteration of deep plexus was also demonstrated by Coscas *et al*. that found at OCTA ischemic areas more frequent in the deep capillary plexus^9^.

Previous *in vivo* animal studies have demonstrated that the venous flow from the deep vascular network joins the major veins in the superficial layers through transverse venules so that when a RVO is present the intravascular pressure increases and the elevation of hydrostatic pressure is more rapid and severe in the deep network resulting in a perfusion decrease in the retinal tissues drained by the deep capillary plexus^11^.

This vessel topographic organization discovered in animals has also been hypothesized by Bonnin *et al*. that studied the vessel organization both in the superficial and in the deep plexus using OCTA in human eyes^12^. He found that the deep capillary plexus is composed of a capillary vortex arrangement, whose centers are aligned along the course of the macular superficial venules thus suggesting that capillary vortex drain into the superficial venules^12^.

Differences in superficial vessel density reduction compared to control eyes between CRVO and BRVO was probably related to a more severe perfusion decrease in BRVO in the affected sectors involving both plexuses. OCTA results confirm baseline characteristics of eyes at FA that showed higher percentage of macular ischemia in BRVO compared to CRVO.

It has been demonstrated that intravitreal administration of anti VEGF drugs in RVOs leads to a progression of retinal ischemia^13^,^14^. In our series there were no significant differences in the percentage of eyes previously treated with anti VEGF thus this aspect cannot explain differences between the two types of RVOs in superficial retinal vessel density. In addition the treatment with anti VEGF was performed more than 9 months prior to the entry in the study.

Retinal vessel density did not change significantly after intravitreal Ozurdex implant in all types of RVOs although retinal macular thickness significantly decreased with concurrent increase of VA and retinal sensitivity at microperimetry.

It has been demonstrated that intravitreal steroid such as dexamethasone or triamcinolone in eyes with RVOs or diabetic retinopathy causes a reduction of arteriolar or venular vessel diameter probably due to a blockage of vascular endothelial growth factor with macular edema improvement^15^,^16^.

Influence of intravitreal steroid treatment on vessel density has not been previously evaluated.

In our study some aspects could have influenced vessel density in the macular area after treatment: disappearance of macular edema and steroid action on vessel diameter.

The presence of macular edema with intraretinal cysts may lead to vessel displacement in the area of interest thus at edema disappearance vessel topographic distribution may change.

In addition as already mentioned vessel caliber could change thus influencing vessel density in an area of interest. At a subjective evaluation we observed a normalization of vessel caliber after retinal edema resolution with deep vessels being less angiectatic nevertheless at a quantitative vessel density analysis we did not find a significant change of vessel density both in the superficial and deep plexuses that were significantly reduced compared to normal controls. This is probably due to the ischemic damage of retinal vessels that do not recover after treatment.

The analysis of choriocapillaris density is a previously unreported result in RVOs that was evaluated in our study.

Other authors analysed choriocapillaris density in vascular pathologies such as diabetic retinopathy (RD) or in age related macular degeneration (AMD)^17^,^18^,^19^. Agemy *et al*. found a reduction of choriocapillaris density in patients affected by DR with greater reduction at increasing disease severity^17^.

A reduction of choriocapillaris density has also been found in early stages of dry AMD^18^,^19^.

In our series we found a reduction of choriocapillaris in CRVO and BRVO compared to controls that returned to normal values after disappearance of macular edema related to intravitreal Ozurdex treatment.

Some studies showed in RVOs an increase of subfoveal choroidal thickness complicated by macular edema compared to controls with a reduction after intravitreal treatment either with steroid implant or anti VEGF^20^,^21^. They hypothesize choroid vessel dilation due VEGF hyper expression. Other authors did not find significant increase of subfoveal choroidal thickness compared to normal eyes^22^.

In our study we did not find significant differences in subfoveal choroidal thickness between diseased and normal eyes.

As far as regard choriocapillaris vessel density we can hypothesize that overlying pathologic retina due to edema could attenuate the OCT signal making regions of the choriocapillaris seem reduced. Other hypothesis could be explored comparing large series of RVOs eyes with or without macular edema to ascertain if hyper expression of VEGF could influence choriocapillaris density.

In this study we also evaluated correlation between the OCTA vessel density and functional parameters such as visual acuity and microperimetry and morphologic parameters such as foveal and parafoveal retinal thickness both in eyes with CRVO and BRVO.

No correlation was found between vessel density and functional parameters.

Retinal superficial and deep vessel density was positively correlated to foveal and parafoveal retinal thickness on the contrary choriocapillaris density was negatively correlated to retinal foveal and parafoveal thickness.

OCTA with SSADA algorithm uses blood flow as intrinsic contrast and detects motion in the blood vessel lumen by measuring the variation in reflected OCT signal amplitude between consecutive cross-sectional scans. OCTA calculates flow index which is the average decorrelation value within a region of interest and vessel density calculated as the percentage area occupied by vessels in a circular ROI, with the blood vessels being defined as pixels having decorrelation values above threshold level. The decorrelation value varies within the capillaries and reaches the saturation value in larger blood vessels^23^. It has been demonstrated that macular edema is related to a hyper expression of VEGF and that greater amount of macular edema is associated to higher intraocular levels of inflammatory cytokines such as VEGF causing a disruption of the blood retinal barrier (BRB) with vessel caliber modification^24^,^25^. In our study the increase in vessel density with increasing macular edema could be attributed to an increase in vessel caliber because vessel density is defined as the percentage area occupied by flow pixels. As already stated RVOs patients with macular edema show areas of vessel density reduction mostly in the deep plexus compared to controls probably due to a perfusion decrease. As also reported by Spaide, sites of macular edema are mainly localized in the deep plexus in regions of reduced or absent flow^26^. Nevertheless independently from vascular non perfused areas, the superficial vessels and mainly the perfused vessels of the deep plexus are dilated due to two main mechanisms: increased blood flow resistance at the level of DCP and vascular dilation related to increased levels of cytokines and growth factors such as VEGF due to inflammation and ischemia^26^,^27^. Macular edema in veno-occlusive diseases has been linked to breakdown of blood-ocular barrier due to increased level of inflammatory cytokines and the amount of macular edema is related to intraocular levels of inflammatory cytokines^24^. It is possible to hypothesize that when a greater amount of macular edema is detected and greater levels of inflammatory cytokines such as VEGF are present they account for vessel dilation measured as a vessel density increase with OCTA in terms of increase of percentage area of vessel density in a ROI of interest.

On the contrary at increasing macular edema choriocapillaris density decreases probably due to the greater masking effect of the overlying thickened retina. In general signal strength of choriocapillaris and choroid is strongly attenuated by RPE and these layers have low signal with conventional spectral domain (SD) OCTA. In addition as already reported by Spaide in his review on image artifacts in OCTA en face imaging that relies on segmentation strategies are going to have difficulty in diseased eyes. In cases of retinal edema, the layers increase in thickness and the signal strength of the underlying choroid is reduced^28^. The reduced signal of SDOCTA limit has been partially overcome by swept source (SS) OCTA due to longer center wavelength that is less attenuated by RPE and less susceptibility to sensitivity roll-off allowing better visualization of choroid and choriocapillaris^29^. Probably the use of SSOCTA would permit also in case of RVO better visualization of choriocapillaris allowing more definitive conclusion on CC changes in case of macular edema.

Functional parameters such as microperimetry and visual acuity were negatively correlated with foveal and parafoveal thickness.

In conclusion our study demonstrated that retinal vessel density is reduced both in CRVO and BRVO eyes with main involvement of the deep plexus and that vessel density does not recover after intravitreal steroid treatment. Choriocapillaris density reduction should be better explored to evaluate the reliability of this finding or attribute it to a limit of OCTA in choriocapillaris analysis in eyes with pathologic overlying retina.

Other limits of this study are related to possible segmentation errors in diseased eyes particularly in presence of macular edema and to vessel density analysis errors due to projection artifacts from inner vascular plexus in the outer retina.

## Materials and Methods

### Study participants

Sixty-three eyes of 63 patients with ophthalmoscopy and fluorescein angiography (FA) diagnosis of either monolateral central retinal vein occlusion (CRVO) or BRVO complicated by macular edema were enrolled in the study. Forty-nine eyes were treatment-naive with recent macular edema (less than 3 months) and 14 eyes were previously treated with anti-VEGF with recurrence of macular edema (last injection over 9 months prior to study entry). Patients consecutively presented at the Department of Ophthalmology, University “G. d’Annunzio” of Chieti-Pescara, between June 2015 and March 2016. Forty age-matched healthy subjects were included as controls. The Institutional Review Board of the Department of Medicine and Science of Aging of University “G. d’Annunzio of Chieti-Pescara, Italy, approved this study and patients signed informed consent to the use of their data. The study adhered to the tenets of the Declaration of Helsinki.

Criteria for inclusion were: 1) age 18 years old; 2) best-corrected visual acuity (BCVA) greater than 0.5 LogMAR in the study eye at baseline examination (to ensure proper execution of examination); 3) confirmed ophthalmologic and fluorescein angiography diagnosis of RVO.

The exclusion criteria were: 1) any ocular surgery (included intravitreal injections) in the study eye in the last 6 months; 2) laser treatment in the study eye; 3) history of glaucoma; 4) media opacity in the study eye.

### Study Protocol

All recruited patients underwent a complete ophthalmic evaluation, including assessment of BCVA, tonometry, slit-lamp biomicroscopy, and indirect fundus ophthalmoscopy.

BCVA was assessed using an Early Treatment Diabetic Retinopathy Study (ETDRS) chart.

Furthermore, all patients were tested by means of XR Avanti® AngioVue OCTA (Optovue Inc., Fremont, CA, USA).

RVO were classified according to the location of the occluded vessel: central retinal vein occlusion (CRVO); branch retinal vein occlusion (BRVO); and hemiretinal vein occlusion (hemi-RVO).

FA images previously acquired with Heidelberg Retina Angiograph *2* HRA2 (HRA+OCT Spectralis: Heidelberg Engineering, Heidelberg, Germany) were used to assess the presence of ischemia in the macular area and RVOs were subjectively graded as ischemic or not by two independent experienced retina specialists (LT and LDA) and a final grade with presence or absence of macular ischemia was formulated if an agreement was reached by the two specialists.

In addition patients willing to improve their visual acuity, with no ocular or systemic contraindications to steroid treatment and signing an informed consent were treated with a sustained-release dexamethasone 0.7 mg intravitreal implant (DEX implant; Ozurdex, Allergan, Inc.) within 7 days from baseline examination.

### Procedures

#### SD-OCT Angiography with XR Avanti

XR Avanti® AngioVue OCTA (Optovue Inc., Fremont, CA, USA) is a device with a high-speed of 70,000 axial scans per second, using a light source of 840 nm, and an axial resolution of 5 μm. The AngioVue OCTA system based on SSADA algorithm (Version: 2015.1.0.90) uses blood flow as intrinsic contrast. Indeed, the flow is detected as a variation over time in the speckle pattern formed by interference of light scattered from RBC and adjacent tissue structure^5^,^30^.

Before imaging, each subject’s pupils were dilated with a combination of 0,5% tropicamide and 10% phenylephrine. Study participants underwent SD-OCT imaging following a protocol that included AngioVue OCT 3D volume set of 3 × 3 mm, consisting of 304 × 304 pixels in the transverse dimension. An internal fixation light was used to center the scanning area.

One FastX (horizontal raster) set and one FastY (vertical raster) set were performed for each acquisition scan. Scans with low quality (i.e. if the subject blinked or if there were significant motion artifacts) were excluded and repeated until good quality was achieved. Three scans for each patient were captured (all with a signal straight index 60) and the scan of best quality was chosen for analysis.

### Vascular layer segmentation

Vascular retinal layers were visualized and segmented based on the default settings of the automated software algorithm of the XR Avanti AngioVue OCTA^10^,^17^. The superficial plexus consists of the capillaries 3 μm below the internal limiting membrane (ILM) to 15 μm below the inner plexiform layer (IPL). The deep plexus extends from 15 μm to 70 μm below the IPL. The choriocapillaris consists of capillaries in a 30 μm thick layer posterior to the retinal pigment epithelium-Bruch membrane junction. The software option to remove projection artifacts from inner vascular plexus in the outer retina was selected. Two observers, independently, checked image quality and excluded poor quality images leading to possible segmentation errors. Eventual manual adjustment of layer segmentation in case of inaccurate segmentation was performed by two-retina specialist (LT and EB) and the manual adjustment of segmentation was chosen only if a consensus was reached.

### Quantitative vessel analysis

Objective quantification vessel density was evaluated on the OCTA en face images for each eye using the AngioVue software (Optovue, Inc., Fremont, CA, USA). The flow area was calculated with a user defined circular region of interest (ROI) and a threshold. The area within the ROI with intensities greater than the threshold was calculated. The vessel density was defined as the percentage area occupied by vessels in a circular ROI centered on the center of the foveal avascular zone and with a diameter of 2.5 mm. The AngioVue software automatically splits the ROI into two fields: the foveal area, a central circle with a diameter of 1 mm; and the parafoveal area that constitutes the remaining part inside the ROI.

The AngioVue software automatically outputs the vessel density percentage inside the foveal area (foveal vessel density – FVD), in the whole parafoveal area (parafoveal vessel density – PFVD), in the hemi superior and inferior fields, and in different quadrants of the parafoveal area (temporal, superior, nasal and inferior). FVD and PFVD of superficial and deep capillary plexuses and of choriocapillaris were analyzed.

The vessel density is calculated using the formula previously described^5^,^30^, as follows:


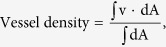


where V is 1 when the OCTA value is above a background threshold and 0 otherwise. A is the area of interest.

For each patient whole enface (foveal and parafoveal) vessel density, foveal vessel density, parafoveal vessel density, superior and inferior hemi parafoveal vessel density in the superficial plexus, deep plexus and in the choriocapillaris were calculated.

### Qualitative vessel analysis

Two independent observers (LT and LDA) subjectively evaluated OCTA in the 3 × 3 mm scan of best quality. Vascular anomalies were evaluated in terms of vessels caliper (regular or irregular such as telangectatic vascular abnormalities and/or microaneurysm), vessel course (regular or irregular such as distorted) and density (normal or rarified).

Perifoveal capillaries were evaluated to disclose disruption or integrity of the perifoveal anastomotic arcades.

### Foveal and parafoveal retinal thickness analysis

Foveal retinal thickness (FRT) and parafoveal retinal thickness (PFRT) in different retinal quadrants were automatically calculated by the software on the OCTA 3 × 3 mm volume scan (XR Avanti®; Optovue, Inc., Fremont, CA, USA) from ILM to RPE (full retinal thickness). A circular ROI centered on the center of the foveal avascular zone with a diameter of 2.5 mm was considered for retinal thickness analysis: central foveal area (1 mm of diameter) and parafoveal area that constitutes the remaining part inside the ROI (total parafoveal area or temporal, superior, nasal and inferior quadrants).

#### Choroidal thickness

Cross-sectional SD-OCT scan (10 mm scan length, 3 mm scan depth) of macular region was performed using the XR Avanti® SD-OCT. The obtained scan provides visualization of structures from deep choroid well into the vitreous, in a single B-scan. The images were shown and measured with the XR Avanti® software. The choroid was manually measured in the sub-foveal (SF) location by a trained investigator (L.T.) from the outer portion of the hyper-reflective line corresponding to the RPE to the inner surface of the sclera.

#### Microperimetry

Microperimetry was performed by means of the MP-1 Microperimeter (Nidek Technologies, Padova, Italy), the latter using an infrared fundus camera with a liquid crystal display software-controlled.

All patients were dilated with tropicamide 1% eye drops and, after a pre-test training; 5 minutes of dark adaptation were performed. The test is routinely carried out with an automated eye tracking system, which provides real-time compensation for eye movements and allows improved presentation of a stimulus at the predefined retinal location. During the test, the patient was encouraged to fix a red ring target, 1° in diameter, on a white monochromatic background at 4 asb. Then, the retinal sensitivity was tested by means of a customized radial grid centered on the fovea and having 77 Goldman III stimuli covering the central 20°. Therefore, the retinal sensitivity can be measured easily because the level of stimulation changes automatically and progressively during the microperimetry test. The stimulus intensity ranged from 0 dB to 20 dB (0 dB corresponded to the strongest signal intensity of 127 cd/m^2^) in 1-dB steps, and the duration of each stimulus was 200 milliseconds. Finally, in order to improve the correlation between microperimetric data with retinal characteristics, results were matched with a color digital retinography obtained with the MP1 color fundus camera. To assess central macular retinal sensitivity, differential light threshold values were compared by calculating the mean of the central 4° and 8°, and the superior and inferior 20° hemi-macular area which was averaged automatically by the MP-1 microperimetry software program of mean sensitivity in a polygon.

#### Treatment

Sustained-release dexamethasone 0.7 mg intravitreal implant (Ozurdex®) was injected in the vitreous cavity. All injections were performed in the operation room, and IVDEX was inserted into the vitreous cavity through the pars plana using a customized, single-use 22-gauge applicator. Patients were treated with a topical ophthalmic antibiotic for 10 days after the treatment.

#### Main outcome measures

Patients were examined at baseline and at 60 days after the IVDEX implant.

Main outcome measures were mean VA; microperimetry at 4°, 8° and 20°; foveal and parafoveal vessel density and retinal macular thickness and subfoveal choroidal thickness.

#### Sample size determination and statistical analysis

The estimation of the number of eyes was based on the main endpoint criteria. A planned sample size of 40 control patients and 20 patients in each of the RVO’s groups was expected to provide 80% power for a two-sided test with significance level of 0.05, assuming an effect size of 3 dB difference of sensibility between case and controls with between subjects’ pooled standard deviation of 4 dB.

The quantitative variables were summarized as mean and standard deviation (SD), qualitative variables as frequency and percentage. A Shapiro-Wilk’s test was performed to evaluate the departures from normality distribution for each variable.

Different two-way analysis of variance (ANOVA) was performed to evaluate the effect of type of occlusion (CRVO and BRVO) and presence of macular ischemia and their interaction on BCVA, microperimetry, macular thickness, choroidal thickness and vessel density parameters. Contrasts analysis, a priori specified were performed to pairwaise comparisons among each of the three RVO groups (CRVO vs BRVO ST, CRVO vs BRVO IT and BRVO ST vs BRVO IT) and control group. Bonferroni- corrected p value were presented.

Chi-square test was applied to assess differences in the proportion between groups.

The Pearson correlation coefficient (R) was performed to analyze the correlation among BCVA, microperimetry, macular thickness and vessel density.

Different linear mixed models for repeated data were applied to regress pre and post treatment measures on the fixed-effect factors assuming unstructured covariance matrix. Linear mixed model for repeated measurements was also used to evaluate the effect of each factor (presence of macular ischemia and treatment) and their interaction on BCVA, microperimetry, macular thickness and vessel density parameters. In all models a priori contrasts, were used to compare mean of different parameters between pre and post treatment.

Statistical analysis was performed using IBM^®^ SPSS Statistics v 20.0 software (SPSS Inc, Chicago, Illinois, USA).

## Additional Information

**How to cite this article**: Rodolfo, M. *et al*. Optical coherence tomography angiography microvascular findings in macular edema due to central and branch retinal vein occlusions. *Sci. Rep.* **7**, 40763; doi: 10.1038/srep40763 (2017).

**Publisher's note:** Springer Nature remains neutral with regard to jurisdictional claims in published maps and institutional affiliations.

## Supplementary Material

Supplementary Table and Figures

## Figures and Tables

**Table 1:** Functional parameters (visual acuity and microperimetry) in patients with retinal vein occlusion at baseline.

Variable	Controls	CRVO	*p-value vs controls*	BRVO ST	*p-value vs controls*	BRVO IT	*p-value vs controls*	*p-value*^a^
Microperimetry (dB)								
20 degree sensitivity	10.55 ± 4.37	7.40 ± 4.23	*0.136*	9.98 ± 3.27	*0.960*	6.91 ± 3.14	***0.052***	*0.125*
8 degree sensitivity	13.41 ± 1.01	5.50 ± 3.56^*,^°	***<0.001***	9.42 ± 2.63^+^	***<0.001***	5.36 ± 2.94	***<0.001***	***<0.001***
4 degree sensitivity	13.02 ± 5.28	3.75 ± 2.20^***^	***<0.001***	8.47 ± 2.10^+++^	***0.007***	3.96 ± 1.73	***<0.001***	***0.025***
Superior emifield	10.55 ± 4.32	7.39 ± 3.92	*0.159*	8.96 ± 4.56	*0.577*	5.87 ± 3.87	***0.011***	***0.035***
Inferior emifield	12.44 ± 1.64	7.45 ± 4.24^*^	***<0.001***	11.38 ± 2.35^+^	*0.450*	7.27 ± 3.32	***<0.001***	***<0.001***
**BCVA (logMar)**	0.05 ± 0.07	0.35 ± 0.12	***<0.001***	0.33 ± 0.29	***<0.001***	0.41 ± 0.28	***<0.001***	*0.594*

CRVO, central retinal vein occlusion; BRVO ST, superotemporal branch retinal vein occlusion; BRVO IT, inferotemporal branch retinal vein occlusion.

^*^p < 0.05; ^**^p < 0.01; ^***^p < 0.001 CRVO *vs* BRVO ST.

^°^p < 0.05; ^°°^p < 0.01; ^°°°^p < 0.001 CRVO *vs* BRVO IT.

^+^p < 0.05; ^++^p < 0.01; ^+++^p < 0.001 BRVO ST *vs* BRVO IT.

^a^Probability that values are influenced by type of occlusion (CRVO and BRVO) adjusted for presence of macular ischemia.

**Table 2:** Retinal and choriocapillaris vessel density in patients with retinal vein occlusion at baseline.

Variable	Controls	CRVO	*p-value vs controls*	BRVO ST	*p-value vs controls*	BRVO IT	*p-value vs controls*	*p-value*^a^
Density of superior plexus (%)								
Whole EnFace	50.06 ± 3.90	47.68 ± 3.24	*0.289*	46.59 ± 3.87	***0.022***	45.81 ± 4.12	***0.011***	*0.282*
Fovea	30.76 ± 7.07	35.43 ± 3.29	*0.299*	32.45 ± 7.96	*0.867*	36.88 ± 11.84	*0.090*	***0.033***
ParaFovea	51.85 ± 4.23	48.47 ± 2.65	*0.089*	47.58 ± 3.83	***0.005***	46.83 ± 3.97	***0.003***	*0.426*
Para-Superior-Hemifield	52.04 ± 4.75	48.59 ± 2.73	*0.134*	45.06 ± 4.77	***<0.001***	48.75 ± 2.50	*0.132*	***0.001***
Para-Inferior-Hemifield	51.67 ± 4.15	48.34 ± 2.84	*0.128*	50.10 ± 4.11^+^	*0.589*	44.94 ± 6.08	***<0.001***	***0.016***
Density of deep plexus (%)								
Whole EnFace	57.46 ± 3.15	48.83 ± 4.61	***<0.001***	52.32 ± 5.30	***0.001***	51.29 ± 6.17	***<0.001***	*0.443*
Fovea	26.78 ± 7.73	25.85 ± 6.54	*0.985*	25.72 ± 5.94	*0.966*	27.54 ± 10.02	*0.991*	*0.713*
ParaFovea	60.32 ± 3.61	50.03 ± 4.92	***<0.001***	55.13 ± 5.92	***0.003***	53.41 ± 6.98	***<0.001***	*0.407*
Para-Superior-Hemifield	60.47 ± 4.30	49.85 ± 5.14	***<0.001***	51.30 ± 6.96	***<0.001***	56.97 ± 6.62	*0.213*	***0.001***
Para-Inferior-Hemifield	60.17 ± 3.53	50.21 ± 6.80	***<0.001***	58.96 ± 5.50^+^	*0.798*	49.87 ± 8.23	***<0.001***	***0.001***
Density of choriocapillaris (%)								
Whole EnFace	66.82 ± 1.43	63.81 ± 1.17^*^	***<0.001***	64.60 ± 1.70	***0.002***	62.6 ± 4.11	***<0.001***	*0.262*
Fovea	66.61 ± 3.57	52.03 ± 15.67^*^	***<0.001***	66.03 ± 5.94	*0.992*	63.31 ± 7.87	*0.482*	*0.115*
ParaFovea	66.34 ± 1.71	59.74 ± 4.51	***<0.001***	64.56 ± 2.48	*0.115*	61.32 ± 4.64	***<0.001***	***0.047***
Para-Superior-Hemifield	66.29 ± 1.93	59.38 ± 3.76^°^	***<0.001***	60.82 ± 5.52^+^	***<0.001***	65.87 ± 3.31	*0.977*	***0.001***
Para-Infererior-Hemifield	66.39 ± 1.80	60.10 ± 6.77^*^	***0.001***	68.35 ± 5.06^++^	*0.386*	56.78 ± 8.62	***<0.001***	***0.001***

CRVO, central retinal vein occlusion; BRVO ST, superotemporal branch retinal vein occlusion; BRVO IT, inferotemporal branch retinal vein occlusion.

^*^p < 0.05; ^**^p < 0.01; ^***^p < 0.001 CRVO *vs* BRVO ST.

^°^p < 0.05; ^°°^p < 0.01; ^°°°^p < 0.001 CRVO *vs* BRVO IT.

^+^p < 0.05; ^++^p < 0.01; ^+++^p < 0.001 BRVO ST *vs* BRVO IT.

^a^Probability that values are influenced by type of occlusion (CRVO or BRVO) adjusted for presence of macular ischemia.

**Table 3:** Morphologic parameters (subfoveal choroidal thickness) in patients with retinal vein occlusion at baseline.

Variable	Controls	CRVO	*p-value vs controls*	BRVO ST	*p-value vs controls*	BRVO IT	*p-value vs controls*	*p-value*^a^
**Subfoveal choroidal thickness (μm)**	257.81 ± 46.29	223.62 ± 71.33	*0.672*	296.10 ± 78.94	*0.242*	271.75 ± 93.43	*0.911*	*0.214*

CRVO, central retinal vein occlusion; BRVO ST, superotemporal branch retinal vein occlusion; BRVO IT, inferotemporal branch retinal vein occlusion.

^a^Probability that values are influenced by type of occlusion (CRVO and BRVO) adjusted for presence of macular ischemia.

**Table 4:** Morphologic parameters (subfofeal choroidal thickness) in patients with retinal vein occlusion after treatment.

	CRVO (n = 10)			BRVO ST (n = 12)			BRVO IT (n = 16)		
	Before treatment	After treatment	*p-value*^a^	Before treatment	After treatment	*p-value*^a^	Before treatment	After treatment	*p-value*^a^
**Subfoveal choroidal thickness (μm)**	223.62 ± 71.33	232.60 ± 62.44	*0.192*	296.10 ± 78.94	313.33 ± 50.82	*0.533*	271.75 ± 93.43	252.50 ± 95.05	*0.140*

CRVO, central retinal vein occlusion; BRVO ST, superotemporal branch retinal vein occlusion; BRVO IT, inferotemporal branch retinal vein occlusion.

^a^Probability that values are influenced by treatment adjusted for presence of macular ischemia.

**Table 5:** Retinal and choriocapillaris vessel density in patients with retinal vein occlusion after treatment.

	CRVO (n = 10)			BRVO ST (n = 12)			BRVO IT (n = 16)		
	Before treatment	After treatment	*p-value*^a^	Before treatment	After treatment	*p-value*^a^	Before treatment	After treatment	*p-value*^a^
Density of sup plexus (%)									
Whole EnFace	47.68 ± 3.24	45.78 ± 5.16	*0.548*	46.59 ± 3.87	47.42 ± 2.41	***0.039***	45.81 ± 4.12	45.63 ± 2.17	*0.199*
Fovea	35.43 ± 3.29	31.37 ± 3.97	***<0.001***	32.45 ± 7.96	31.28 ± 7.60	***0.036***	36.88 ± 11.84	36.06 ± 9.71	*0.086*
ParaFovea	48.47 ± 2.65	49.60 ± 4.87	*0.334*	47.58 ± 3.83	47.03 ± 2.49	***0.039***	46.83 ± 3.97	46.91 ± 2.12	*0.097*
Para-Superior-Hemifield	48.59 ± 2.73	49.89 ± 4.54	*0.212*	45.06 ± 4.77	45.72 ± 2.31	***0.009***	48.75 ± 2.50	48.27 ± 2.69	*0.164*
Para-Inferior-Hemifield	48.34 ± 2.84	49.33 ± 5.26	*0.649*	50.10 ± 4.11	51.33 ± 2.66	***0.040***	44.94 ± 6.08	45.49 ± 2.33	*0.166*
Density of deep plexus (%)									
Whole EnFace	48.83 ± 4.61	49.93 ± 4.95	*0.198*	52.32 ± 5.30	52.82 ± 0.87	***0.038***	51.29 ± 6.17	51.40 ± 4.82	***0.027***
Fovea	25.85 ± 6.54	26.13 ± 6.75	*0.155*	25.72 ± 5.94	26.92 ± 4.45	***0.036***	27.54 ± 10.02	27.37 ± 5.10	***0.014***
ParaFovea	50.03 ± 4.92	52.42 ± 6.04	*0.146*	55.13 ± 5.92	56.26 ± 2.37	***0.039***	53.41 ± 6.98	53.56 ± 5.59	*0.366*
Para-Superior-Hemifield	49.85 ± 5.14	51.69 ± 6.49	***0.067***	51.30 ± 6.96	51.92 ± 1.22	***0.038***	56.97 ± 6.62	56.15 ± 4.73	*0.875*
Para-Inferior-Hemifield	50.21 ± 6.80	53.15 ± 5.97	*0.490*	58.96 ± 5.50	57.58 ± 3.56	***0.039***	49.87 ± 8.23	50.83 ± 6.79	*0.053*
Density of choriocapillaris (%)									
Whole EnFace	63.81 ± 1.17	66.36 ± 1.79	***0.005***	64.60 ± 1.70	65.27 ± 1.76	***0.042***	62.6 ± 4.11	65.57 ± 1.50	*0.152*
Fovea	52.03 ± 15.67	65.51 ± 3.56	***0.011***	66.03 ± 5.94	70.36 ± 4.30	***0.039***	63.31 ± 7.87	63.69 ± 2.96	*0.071*
ParaFovea	59.74 ± 4.51	66.13 ± 1.88	***<0.001***	64.56 ± 2.48	66.83 ± 0.67	***0.042***	61.32 ± 4.64	65.21 ± 1.63	***0.009***
Para-Superior-Hemifield	59.38 ± 3.76	66.25 ± 1.35	***<0.001***	60.82 ± 5.52	64.32 ± 2.63	***0.041***	65.87 ± 3.31	65.30 ± 1.49	*0.153*
Para-Inferior-Hemifield	60.10 ± 6.77	66.02 ± 2.46	*0.072*	68.35 ± 5.06	69.32 ± 3.91	***0.042***	56.78 ± 8.62	65.11 ± 1.80	***0.023***

CRVO, central retinal vein occlusion; BRVO ST, superotemporal branch retinal vein occlusion; BRVO IT, inferotemporal branch retinal vein occlusion.

^a^Probability that values are influenced by treatment adjusted for presence of macular ischemia.
