# Corrigendum: Optical coherence tomography angiography microvascular findings in macular edema due to central and branch retinal vein occlusions

**DOI:** 10.1038/srep42570

**Published:** 2017-02-23

**Authors:** Rodolfo Mastropasqua, Lisa Toto, Luca Di Antonio, Enrico Borrelli, Alfonso Senatore, Marta Di Nicola, Giuseppe Di Martino, Marco Ciancaglini, Paolo Carpineto

Scientific Reports 7: Article number: 4076310.1038/srep40763; published online: 01 18 2017; updated: 02 23 2017

The original version of this Article contained errors in the spelling of authors Rodolfo Mastropasqua, Lisa Toto, Luca Di Antonio, Enrico Borrelli, Alfonso Senatore, Marta Di Nicola, Giuseppe Di Martino and Marco Ciancaglini, which were incorrectly given as Mastropasqua Rodolfo, Toto Lisa, Di Antonio Luca, Borrelli Enrico, Senatore Alfonso, Di Nicola Marta, Di Martino Giuseppe, Ciancaglini Marco respectively.

These errors have now been corrected in the HTML and PDF versions of this Article.

