# Supplementary material for: Optical coherence tomography angiography microvascular findings in macular edema due to central and branch retinal vein occlusions
Source: Sci Rep. 2017 Jan 18;7:40763. doi: 10.1038/srep40763 (PMC5241684; doi:10.1038/srep40763)
Supplement: Supplementary Table and Figures [file srep40763-s1.pdf]

Optical coherence tomography angiography microvascular findings in macular edema due to central and branch retinal vein occlusions

Mastropasqua Rodolfo,[1+] Toto Lisa,[2*+] Di Antonio Luca,[2] Borrelli Enrico,[2] Senatore Alfonso,[2] Di Nicola Marta,[3] Di Martino Giuseppe,[3] Ciancaglini Marco,[4] Paolo Carpineto [2]

[1]Ophthalmology Clinic, Moorfields Eye Hospital, London, EC1V 2PD, UK

[2]Ophthalmology Clinic, Department of Medicine and Science of Ageing, University G. D'Annunzio Chieti-Pescara, Chieti, 66100, Italy

[3] Department of Experimental and Clinical Sciences, Laboratory of Biostatistics, University "G. d'Annunzio" Chieti-Pescara, Chieti, 66100, Italy

[4] Ophthalmology Clinic, University of l'Aquila, Aquila, 67100, Italy

[+]The first two authors equally contributed to the manuscript and should be considered as co-first authors

*** Corresponding Author:**

Lisa Toto

Email: l.toto@unich.it

None of the authors has any proprietary/financial interest to disclose

Table S1. Morphologic parameters (macular thickness) in patients with retinal vein occlusion at baseline.

Variable	Controls	CRVO	*p-value vs controls*	BRVO ST	*p-value vs controls*	BRVO IT	*p-value vs controls*	*p-value*[a]
**Macular thickness (ILM-RPE) (μm)**								
Fovea	251.11±39.67	575.25±111.77[**]	***<0.001***	373.58±125.47	***<0.001***	465.89±145.35	***<0.001***	***0.034***
ParaFovea	317.42±17.44	451.63±66.78	***<0.001***	391.33±73.90	***<0.001***	420.78±81.96	***<0.001***	*0.493*
Para-Temporal	307.82±18.49	450.63±74.29	***<0.001***	395.00±86.96	***<0.001***	438.33±94.93	***<0.001***	*0.493*
Para-Superior	314.16±50.16	452.63±72.95	***<0.001***	446.83±127.25	***<0.001***	347.56±26.42	*0.465*	***0.001***
Para-Nasal	321.45±19.84	473.38±75.31[*]	***<0.001***	378.92±55.02	***0.002***	404.11±90.50	***<0.001***	*0.074*
Para-Inferior	318.79±17.30	429.63±57.45	***<0.001***	345.25±51.10[++]	*0.493*	493.33±150.61	***<0.001***	***<0.001***

CRVO, central retinal vein occlusion; BRVO ST, superotemporal branch retinal vein occlusion; BRVO IT, inferotemporal branch retinal vein occlusion;
ILM, inner limiting membrane; RPE, retinal pigment epithelium; IPL, inner plexiform layer.
[*] p<0.05; [**] p<0.01; [***] p<0.001 CRVO *vs* BRVO ST
[°] p<0.05; [°°] p<0.01; [°°°] p<0.001 CRVO *vs* BRVO IT
[+] p<0.05; [++] p<0.01; [+++] p<0.001 BRVO ST *vs* BRVO IT
[a] Probability that values are influenced by type of occlusion (CRVO and BRVO) adjusted for presence of macular ischemia.

Table S2. Functional parameters (visual acuity and microperimetry) in patients with retinal vein occlusion after treatment.

Variable	CRVO (n=10)			BRVO ST (n=12)			BRVO IT (n=16)		
	Before treatment	After treatment	*p-value*[a]	Before treatment	After treatment	*p-value*[a]	Before treatment	After treatment	*p-value*[a]
**Microperimetry (dB)**									
20 degree sensitivity	7.39±3.92	12.04±1.45	***0.003***	8.96±4.56	10.17±0.36	*0.313*	5.87±3.87	8.88±4.58	***0.014***
8 degree sensitivity	7.45±4.24	10.16±2.27	***0.001***	11.38±2.35	13.33±1.81	*0.130*	7.27±3.32	11.33±2.32	***0.041***
4 degree sensitivity	7.40±4.23	10.76±1.98	***<0.001***	9.98±3.27	11.20±1.08	*0.181*	6.91±3.14	10.00±3.50	***0.018***
Superior emifield	5.50±3.56	11.32±2.18	***<0.001***	9.42±2.63	11.30±1.70	*0.215*	5.36±2.94	9.10±3.87	***0.013***
Inferior emifield	3.75±2.20	11.86±3.14	***<0.001***	8.47±2.10	12.60±0.62	***<0.001***	3.96±1.73	7.75±2.93	***0.017***
**BCVA (logMar)**	0.35±0.12	0.10±0.09	***<0.001***	0.33±0.29	0.50±0.09	***0.001***	0.41±0.28	0.15±0.12	***0.003***

CRVO, central retinal vein occlusion; BRVO ST, superotemporal branch retinal vein occlusion; BRVO IT, inferotemporal branch retinal vein occlusion.

[a] Probability that values are influenced by treatment adjusted for presence of macular ischemia

Table S3. Morphologic parameters (macular thickness) in patients with retinal vein occlusion after treatment

	**CRVO (n=10)**			**BRVO ST (n=12)**			**BRVO IT (n=16)**		
	**Before treatment**	**After treatment**	***p-value***[a]	**Before treatment**	**After treatment**	***p-value***[a]	**Before treatment**	**After treatment**	***p-value***[a]
**Macular thickness (ILM-RPE) (μm)**									
Fovea	575.25±111.77	272.20±45.74	***<0.001***	373.58±125.47	258.00±26.35	***0.028***	465.89±145.35	290.50±89.70	***0.015***
ParaFovea	451.63±66.78	329.40±18.86	***<0.001***	391.33±73.90	328.33±48.67	***0.027***	420.78±81.96	334.75±40.65	*0.057*
Para-Temporal	450.63±74.29	311.00±10.69	***0.001***	395.00±86.96	323.00±62.82	***0.034***	438.33±94.93	351.75±81.84	***0.036***
Para-Superior	452.63±72.95	337.00±19.74	***<0.001***	446.83±127.25	333.67±67.67	***0.023***	347.56±26.42	341.50±34.23	*0.074*
Para-Nasal	473.38±75.31	335.80±31.90	***<0.001***	378.92±55.02	339.00±49.97	***0.021***	404.11±90.50	326.75±19.70	*0.101*
Para-Inferior	429.63±57.45	333.80±15.92	***<0.001***	345.25±51.10	317.33±13.87	*0.155*	493.33±150.61	320.00±48.09	*0.054*

CRVO, central retinal vein occlusion; BRVO ST, superotemporal branch retinal vein occlusion; BRVO IT, inferotemporal branch retinal vein occlusion.

[a]Probability that values are influenced by treatment adjusted for presence of macular ischemia

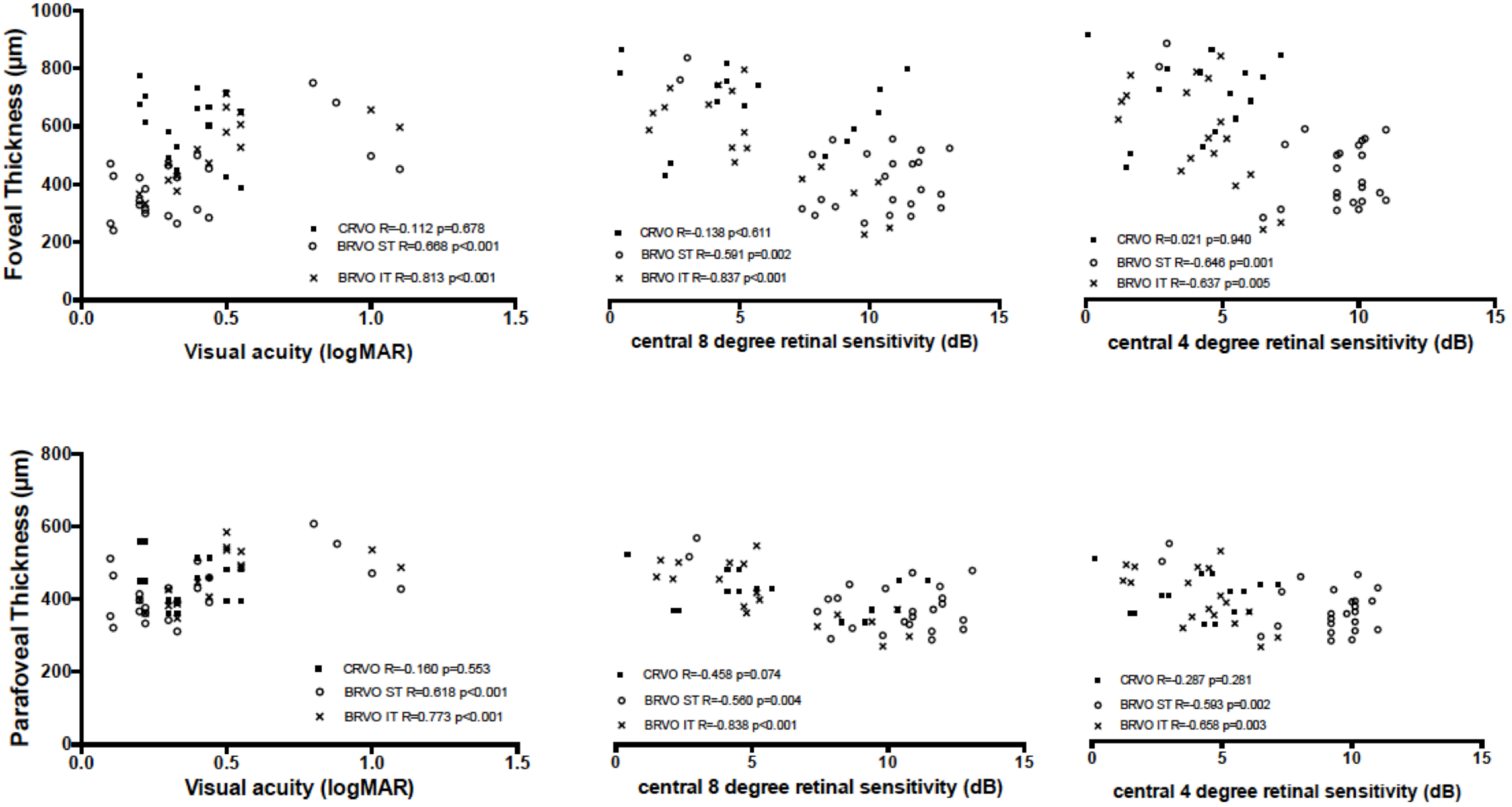

Figure 1S. Correlations [Pearson correlation coefficient (R)] between morphological (foveal and parafoveal thickness) and functional parameters (visual acuity and microperimetry at 4 and 8 degree) in RVOs patients.
